# Changes in the degree of substitution of HES in vivo and their influence on methods for determining HES concentrations in plasma

**DOI:** 10.1371/journal.pone.0290339

**Published:** 2023-08-22

**Authors:** Peter Lukasewitz, Philipp Rischer, Nina Schedel, David Stay, Hinnerk Wulf, Thomas Stief, Christian Volberg

**Affiliations:** 1 Department of Anaesthesiology and Intensive Care Medicine, Philipps University of Marburg, University Hospital Marburg, Marburg, Germany; 2 Institute of Laboratory Medicine, Philipps University of Marburg, University Hospital Marburg, Marburg, Germany; University of Veterinary and Animal Sciences, PAKISTAN

## Abstract

**Objective:**

The degree of substitution (DS) of HES describes the average proportion of substituted glucose molecules in a starch molecule. Although no quantitative studies of the in vivo behavior of the DS have been conducted so far, most pharmacokinetic studies to date have measured HES concentrations using the enzymatic method. This method assumes that at any point in time after an infusion, the DS in a serum remains constant and is identical to the DS in the infused solution. In the present study, we examined the changes in the DS of HES 130/0.42 in vivo in an animal model and compared two methods of measuring HES concentrations in plasma (the enzymatic and the o-Toluidine method).

**Methodology:**

We randomized 22 pigs into 2 groups. After induction of anesthesia, the pigs received 500 ml or 1000 ml of HES 130/0.42 (Tetraspan®). The DS was measured directly after the infusion, then after 30, 60, 120 and 240 minutes. In determining the DS, the hydroxyethyl starch was extracted from the plasma and hydrolyzed with hydrochloric acid to form non-substituted glucose and hydroxyethyl glucose. Subsequently, the concentration of free unsubstituted glucose was determined enzymatically and the total concentration of all (i.e., substituted and unsubstituted) glucose molecules was determined using the o-Toluidine method. From this, the concentration of the substituted glucose (hydroxyethyl glucose) and the DS could be calculated. In addition, the HES concentration was measured first in vitro and then in vivo at any point after the infusion by both the enzymatic method and the o-Toluidine method.

**Results:**

The DS increased significantly directly after the infusion from 0.42 to 0.53 (for 500ml) or to 0.50 (for 1000ml); 4 hours later this had further increased to 0.55 and 0.54, respectively (p <0.0001).

The HES concentration in vitro showed no significant difference (p = 0.17) when determined with the enzymatic and the o-Toluidine method.

In contrast, the serum concentrations in vivo displayed significant differences (p<0.0001) between the two measurement methods. Immediately after the infusion of 500ml HES, the concentration measured with the o-Toluidine method was 31% higher than the one measured with the enzymatic method; 4 hours later, this discrepancy was still at 25%. For 1000 ml HES, the differences amounted to 16% and 25%, respectively.

**Conclusion:**

The DS of HES in vivo increases significantly over time. As a result, an HES concentration measured with the enzymatic method in vivo will be significantly lower than the same concentration determined with the o-Toluidine method.

In future pharmacokinetic studies, HES concentrations should be measured using a method that takes into account changes in the DS in vivo.

## Introduction

**Fig 1:**
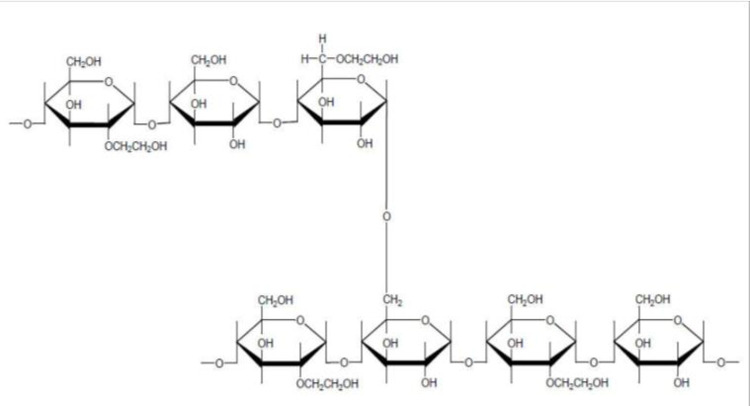
Hydroxyethyl starch (HES) structural formula.

The degree of substitution (DS), along with the C2/C6 ratio and the mean molecular weight, is one of the most important pharmacological characteristics of hydroxyethyl starch (HES). The degree of substitution describes the average number of hydroxyethyl residues per glucose unit. A degree of substitution of 0.5 means that 50% of the glucose units in a starch molecule are substituted with at least one hydroxyethyl group. Hydroxyethyl groups can be introduced only at 3 positions–on carbon atoms 2, 3 and 6 of the glucose unit [1]. All HES specifications are in principle mixtures of partly very dissimilar HES molecules, which differ significantly from one another in terms of molecular size, branching and substitution (Fig 1). When classifying HES, only the statistical mean values such as the mean molecular weight (MW) and the mean degree of substitution are typically specified. The polydispersity of hydroxyethyl starch in terms of molecular weight and degree of substitution has a significant influence on its pharmacokinetics. The smallest molecules, below the renal threshold, are already filtered glomerularly during infusion, the larger ones are continuously enzymatically hydrolyzed to smaller fragments. As a result, the molecular weight distribution in vivo becomes increasingly narrower over time and the average molecular weight increasingly smaller [2,3]. The drop in mean molecular weight in vivo is considerable, especially in the first two to three hours after the infusion—from 130,000 D to 60,000–80,000 D, in the case of HES 130 / 0.4. Only after about three hours does the rate of change decrease significantly [3,4]. The mean molecular weight in vivo is therefore a dynamic, time-dependent variable, driven by enzymatic degradation, renal elimination, and tissue uptake.

In contrast to the mean molecular weight, the in vivo changes of which have been carefully examined for all HES specifications, there is so far no data on the in vivo behavior of the degree of substitution. To date, we do not know how the degree of substitution changes in vivo and which HES molecules remain the longest in the intravascular space. Substitution with hydroxyethyl groups significantly delays the enzymatic hydrolysis of starch. It is known that α-amylase can only hydrolyze glycosidic bonds of unsubstituted glucose units [5]. A lower degree of substitution denotes a larger number of unsubstituted regions in an HES molecule that are more easily accessible to enzymatic degradation. It must therefore be assumed that, within individual HES specifications, it is primarily the lower-substituted molecules and molecular fragments that are preferentially cleaved and that the number of higher-substituted molecules increases proportionally over time.

The method of determining the degree of substitution in vitro was described as early as 1973 [1]. This method assumes that for any HES solution whose degree of substitution is to be established, its concentration is known. Sulfuric acid is added to a defined amount of hydroxyethyl starch and hydrolyzed in a boiling water bath. After hydrolysis, a mixture of glucose and hydroxyethyl glucose molecules is formed. After cooling, the sample is neutralized, and the glucose is subsequently determined enzymatically using the glucose oxidase-peroxidase method. Control experiments have shown that only the unsubstituted, free glucose reacts with the glucose oxidase—peroxidase or hexokinase—glucose-6-phosphate-dehydrogenase system [1]. The degree of substitution is then calculated from the concentration of unsubstituted glucose thus measured and the known HES concentration, i.e. from the amount of all substituted and unsubstituted glucose molecules. However, the Banks method does not allow to investigate changes in the degree of substitution in vivo whenever the HES concentration in the plasma is unknown.

In the present study, the concentration of all HES molecules was hence measured using the o-toluidine method [6]—in addition to the above-described enzymatic determination of the concentration of the unsubstituted HES part. In the o-toluidine method, the amino group of o-toluidine reacts with the carbonyl group (aldehyde group) of the sugar. The aldehyde group is situated on the carbon atom C1, to which no hydroxyethyl groups are bound. This means that the determination of glucose with o-toluidine is not influenced by substitution–when using the o-toluidine method, all glucose molecules are captured [7]. By comparing the enzymatic method, through which the concentration of unsubstituted glucose molecules is measured, with the o-toluidine method, through which both unsubstituted and substituted glucose molecules are recorded, the in vivo changes in the degree of substitution for HES can be determined.

Our hypothesis is: the degree of substitution of HES in the intravascular space increases significantly over time.

## Materials and methods

The study was approved by the responsible authority (Veterinary Department of the Giessen Regional Council, application 49–2011).

This study was a randomized parallel group design with a single dose and two groups of 11 pigs from the "German country pig" breed. Random allocation was performed before each experiment using tables of random numbers. The test animals were female, between 4 and 6 months old and had a weight of 31.7 ± 3.0 kg—group 1 and 33.0 ± 5.0—group 2, respectively (p = 0.45).

After 12 hours of food abstinence the animals were premedicated with ketamine (20 mg/kg, IM), atropine (0.03 mg/kg, IM) and diazepam (1 mg/kg, IM). This was followed by intravenous anesthesia induction with sufentanil (1 μg/kg) and propofol (3 mg/kg). The pigs were then orally intubated and mechanically ventilated with an FiO2 of 0.25 throughout the experiment. After induction the animals received either 500 ml (group1) or 1000 ml (group 2) of HES 130/0.42 (Tetraspan®, B. Braun Melsungen, Germany) at a constant rate over a period of 30 minutes. The dosage 1000 ml at an average weight of animals 32.5 kg BW corresponds exactly to a maximum daily dose of HES 130/0.42 in humans 30 ml/kg BW. We wanted to check whether the dynamics of changes in the degree of substitution is different at higher doses compared to low doses. The anesthesia was maintained with sufentanil (1–5 μg/kg/h) and propofol (2–3 mg/kg/h). Blood samples were taken before commencing infusion, immediately after the end of infusion (0) and 30, 60,120 and 240 min after its end.

Before the actual experiment, two in vitro measurements were carried out. In the first experiment, it was to be established whether the enzymatic glucose determination method indeed only records unsubstituted glucose molecules. To this end, 0.2 ml (12 mg) of hydroxyethyl starch were laced directly with 4.9 ml of 2N hydrochloric acid. They were placed into a boiling water bath for 50 min and subsequently neutralized with 4.9 ml of 2 N NaOH. The glucose concentration was then measured.

In the second experiment, plasma samples, stemming from the blood of the pigs before the infusion, were placed in an ice bath for 1 hour. They were then mixed with hydroxyethyl starch (0.8 ml plasma + 0.2 ml HES, respectively) and the HES concentration was checked immediately using the enzymatic and the o-toluidine methods The aim of this experiment was to detect any in vitro differences between the two methods of measurement.

For detecting the unsubstituted portion of the hydroxyethyl starch, a modified polysaccharide measurement was used [7]. Immediately after centrifugation, the plasma samples (1 ml each) were laced with 0.5 ml of 35% potassium hydroxide solution and boiled in a water bath for 60 minutes. Hydroxyethyl starch was precipitated using absolute ethanol. For this purpose, each of the samples was mixed with 12 ml of alcohol, placed in an ice bath for 60 minutes and then centrifuged at 3000 rpm for 60 minutes. The supernatant was tipped off. The precipitate was hydrolyzed to glucose and hydroxyethylglucose with 5 ml of 2N hydrochloric acid in a boiling water bath for 50 min. After cooling, the samples were neutralized with 5 ml of a 2N NaOH solution and filtered into 10 ml flasks.

Finally, the concentration of unsubstituted glucose molecules was determined enzymatically using hexokinase and glucose-6-phosphate dehydrogenase. The total glucose concentration was measured by means of the o-toluidine method. In the o-toluidine method, 0.5 ml of solution from the 10-ml vials was mixed in a test tube with 5 ml of a 0,6 M o-toluidine solution in acetic acid (o-toluidine for use in the colorimetric determination of glucose, Sigma-Aldrich®, T1199) and boiled in a water bath for 20 minutes. The reaction between o-toluidie in concentrated hot acetic acid produces a green dye. After cooling, the absorbance was measured at 405 nm.

From the start, 0.2 ml of HES 130/0.42 and 0.8 ml of distilled water were used by default in all analytical approaches.

The total glucose (glucose and hydroxyethyl glucose) recorded with the o-toluidine method corresponds to the HES concentration. In addition, the concentration of unsubstituted glucose was translated into HES concentration (as in the common enzymatic method). This made the two methods of measuring HES concentration comparable.

### Statistics

The statistical calculation of the changes in the degree of substitution was carried out independently in each group (500 ml and 1000 ml) using a one-way analysis of variance.

Statistical group comparison of two methods regarding the HES concentration was carried out using the two-way analysis of variance (ANOVA) with repeated measurements.

p <0.05 was assumed to be statistically significant.

## Results

The hydrolysis of HES 140 / 0.42 with hydrochloric acid in vitro and subsequent determination of the glucose concentration were carried out in a test series with 10 samples. The HES concentration in the samples was originally 120 mg/dl (0.2 ml 6% HES + 4.9 ml HCl + 4.9 NaOH). With a degree of substitution of 0.42, this corresponds to a concentration of unsubstituted glucose molecules of 69.6 mg/dl (58%). The enzymatically measured average glucose concentration after in vitro hydrolysis was 70.02 (± 0.275) mg/dl in our experiment, which corresponds to a proportion of exactly 58.35%.

In the second in vitro experiment, in which the enzymatic and o-toluidine methods were compared (22 samples in total), the initial HES concentration was equally 12 mg/ml (0.8 ml plasma + 0.2 ml 6% HES). The direct in vitro comparison showed no significant difference (p = 0.17) between the two methods. The average concentration using enzymatic detection was 11.67 mg/ml (± 0.14) and 11.75 mg/ml (± 0.16) using the o-toluidine method.

In contrast, the serum concentrations in vivo showed significant differences (p <0.0001) between the two groups at every point in time during the experiment.

**Fig 2:**
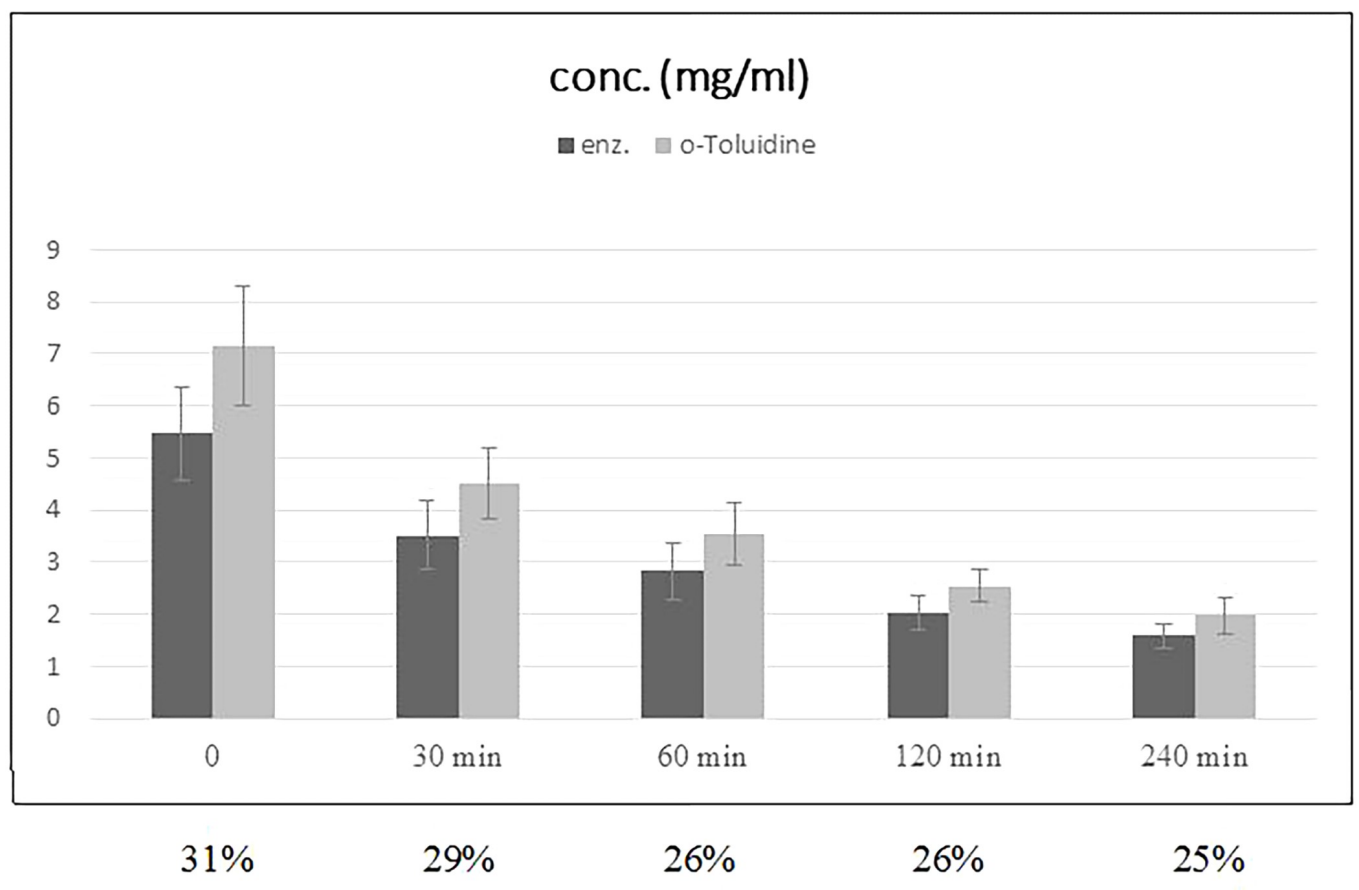
Mean plasma concentration of HES after infusion of 500 ml HES 130/0.42, concentration differences in %.

**Fig 3:**
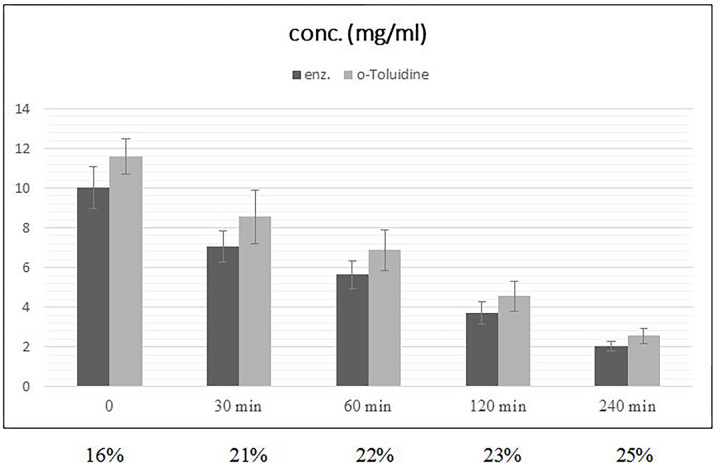
Mean plasma concentration of HES after infusion of 1000 ml HES 130/0.42, concentration differences in %.

The concentration measured with o-toluidine was 31% higher directly after the infusion of 500 ml HES and 25% higher after 4 hours compared to the enzymatic method. After 1000 ml of HES, the differences were 16% and 25%, respectively (Figs 2 and 3).

The degree of substitution corresponds to the ratio of the concentration of the substituted glucose molecules to the total glucose concentration. The concentration of substituted glucose equates to the difference between the total concentration (o-toluidine) and the unsubstituted portion (enzymatic method):

$$Degree\;of\;substitution=\frac{glucose\;conc.(o-toluidine)-glucose\;conc.(enz.)}{glucose\;conc.(o-toluidine)}$$

The degree of substitution increased significantly directly after the infusion from 0.42 to 0.53 and 0.5, respectively, and 4 hours later to 0.55 and 0.54 (p <0.0001). The DS changes are shown in Table 1.

**Table 1:** Changes in the degree of substitution, DS of infusion solution 0,42.

Time after Infusion (min)					
DS	0	30	60	120	240
500 ml	0.53	0.53	0.54	0.53	0.55
±	0,04	0,04	0,03	0,03	0,02
1000 ml	0.50	0.53	0.52	0.53	0.54
±	0,03	0,03	0,03	0,03	0,02

The increase in the degree of substitution from the end of the infusion to the end of the experiment was no longer significant in the 500 ml group (from 0.53 to 0.55) (p = 0.68). After the infusion of 1000 ml, however, the degree of substitution increased significantly over the same period, from 0.50 to 0.54 (p <0.001).

## Discussion

The outcome observed–the significant increase in the degree of substitution of HES in vivo–is not surprising. It can be assumed that it is primarily the low-substituted HES molecules that are hydrolyzed and eliminated and that the degree of substitution in plasma increases over time.

This is supported by the fact that the elimination of HES slows down over time. Although the mean molecular weight is known to decrease in vivo, an increasingly smaller proportion of HES was eliminated per given time unit. In the first hour after the infusion of 500 ml, the HES concentration fell by 50.5% (using the o-toluidine method), in the second hour by 28.2% and in the next two hours by a total of 22%. The decelerating elimination of HES is hence likely related to the slower cleavage of the increasingly larger proportion of more highly substituted molecules.

The more interesting result of the study is the divergence of HES concentrations determined by the two different methods. It stems from the fact that changes in the SD in vivo influence the accuracy of the prevalent enzymatic method of determining the HES concentration. It is astonishing that this has not been considered in pharmacokinetic studies so far.

In the first years after the approval of hydroxyethyl starch as a volume substitute (1973 in the USA and 1974 in Germany), the in vivo concentration of HES in serum and in urine were examined using the Anthrone method for determining carbohydrates [8,9]. With this method, both mono- and polysaccharides were recorded at the same time. The free glucose present in the blood and included in the detection was hence determined separately and then subtracted from the total concentration of carbohydrates. However, with this method, it was extremely difficult to reliably detect very small amounts of polysaccharides in the plasma [7].

In 1981, Förster modified Banks’ method for the specific enzymatic measurement of HES concentration [1,7]. Förster’s method established itself very quickly and has been used, with minor modifications, in most pharmacokinetic studies of all HES generations—HES 450/0.7; 200/0.5, 200/0.62; 70/0.5; 130/0.4 [2,3,10]. The method generally consists of four steps: separation of proteins and other organic components, precipitation of HES, hydrolysis to glucose or hydroxyethyl glucose and quantitative enzymatic determination of the concentration of unsubstituted glucose with glucose oxidase / peroxidase or hexokinase / glucose-6-phosphate dehydrogenase. The concentration of the unsubstituted HES fraction is then translated to the total HES concentration. The reference value represents the unsubstituted portion of the infused solution, which is kept as a standard sample. It is assumed that at every point in time after the infusion, the degree of substitution in the examined plasma samples is identical to the degree of substitution in the standard sample (the infused solution). In other words, the enzymatic method is only correct provided that the degree of substitution remains unchanged in vivo. Since, as we were able to determine, the degree of substitution increases significantly in vivo, the HES concentration measured with the enzymatic method was always lower than when measured with the o-toluidine method. The significant difference (p <0.0001) between the two methods could already be determined at the end of the infusion and persisted throughout the study period.

We would like to illustrate our explanation for why the enzymatic method shows values ​​that are too low and how the difference between the two methods comes about using a simple example:

*Let us take an infused solution with a degree of substitution of 0.4 (40% substituted and 60% unsubstituted glucose molecules), and an enzymatically measured glucose concentration (i.e. the unsubstituted portion), of 3 mg/ml after 2 hours*.

*Option 1*: *The SD remains unchanged in vivo*, *as previously assumed*: *In this case the concentration of 3 mg/ml represents 60% of the total concentration*. *The total HES concentration is therefore 5 mg/ml (regardless of the method of determination)*.
*Option 2*: *The SD increases–e*.*g*., *to 0*.*5 after 2 h*:

*The enzymatic method would not take this SD increase into account—the infused solution would still provide the reference value with an SD of 0.4, i.e. the concentration would remain at 5 mg/ml. In reality, however, the enzymatically measured glucose concentration of 3 mg/ml would, with an SD of 0.5, not represent 60% but only half of the actual total concentration. Using the o-toluidine method, on the other hand, which registers all glucose molecules, the HES concentration measured would be the correct 6 mg/ml–a difference of 20%*.

We would like to emphasize that no significant differences between the two methods could be found in the in vitro test. Only the changes in the degree of substitution in the intravascular space led to the considerable difference between the enzymatic and the o-toluidine method.

In our opinion, there is therefore no doubt that the degree of substitution increases over time. This means that the enzymatically measured HES concentrations in previous pharmacokinetic studies are likely incorrect, as they are too low.

What does this mean for clinical practice?

While it can be assessed positively that the mean molecular weight of HES decreases over time and that in this regard, the solution becomes increasingly uniform in vivo, the increase in the degree of substitution should be viewed rather critically. It is well known that a higher degree of substitution is associated with a longer half-life and a higher rate of complications.

In the perioperative phase with non-septic patients, the SD increase does not seem to have any major clinical relevance.

In 2013, van der Linden [11] showed in a meta-analysis (59 studies with over 4500 patients) that using HES 130/0.4 (waxy maize starch) or HES 130/0.42 (potato starch) intraoperatively with a dosage of 10 to 30 ml/kg does not cause any adverse effects in relation to kidney function (absolute serum creatinine concentration, need for renal replacement therapy), blood loss (erythrocyte transfusion) or mortality compared to other treatment options (crystalloids or gelatin, also in combination with vasoactive substances). Included in the analysis were major abdominal, orthopedic, and cardiac surgery interventions. Other meta-analyses and more recent larger controlled randomized studies confirm this result [12,13].

Likely more problematic are repeated applications in critically ill intensive care patients, especially in septic patients with pre-existing renal dysfunction. The large clinical studies of the last few years showed that in this group of patients, repeated administration of HES, both HES 130/0.42 and 130/0.4, had a negative effect on coagulation and kidney function in the course of therapy [14,15].

Using the enzymatic method, the study of test persons showed almost complete elimination of HES 130/0.4 from the intravascular space after 24 hours, even in the case of mild to severe renal insufficiency [16]. However, it cannot be ruled out that a higher substituted fraction exists that is difficult to eliminate and not detectable using the enzymatic method. With repeated applications, those fractions could accumulate and promote the occurrence of the above-mentioned undesirable side effects.

Unfortunately, there has not been a single clinical study to date examining the complications of HES in relation to serum concentration. Although the infused amount of colloid is known in all studies when included, the concentrations are certainly very different in the case of various kidney and liver dysfunctions. Due to the continuous changes in the mean molecular weight and the degree of substitution in vivo, the mathematical calculation of the kinetics of HES using traditional pharmacokinetic models is made considerably more difficult and can only be used with reservations.

It is unknown as of what amount and serum concentration of HES serious clinical complications are to be expected. We therefore believe that future studies with hydroxyethyl starch should also measure the colloid concentration–using a method that considers changes in the degree of substitution in vivo.
